# Validation of the International Classification of Diseases, Tenth Revision—Clinical Modification Diagnostic Code for Essential Tremor

**DOI:** 10.5334/tohm.905

**Published:** 2024-07-04

**Authors:** Susanna D. Howard, Shikha Singh, Dominick Macaluso, Iahn Cajigas, Whitley W. Aamodt, John T. Farrar

**Affiliations:** 1 Department of Neurosurgery, University of Pennsylvania, Philadelphia, PA, USA; 2 University of Pennsylvania Perelman School of Medicine, Philadelphia, PA, USA; 3 Department of Neurology, University of Pennsylvania, Philadelphia, PA, USA; 4 Department of Biostatistics and Epidemiology, Perelman School of Medicine, University of Pennsylvania, Philadelphia, US

**Keywords:** essential tremor, code validation, claims data, health outcomes research

## Abstract

**Background:**

The positive predictive value (PPV) of the *International Classification of Diseases, Ninth Revision—Clinical Modification* (ICD-9-CM) code for “essential and other specified forms of tremor” in identifying essential tremor (ET) cases was found to be less than 50%. The ability of the ICD-10-CM G25.0 code for “essential tremor” to identify ET has not been determined. The study objective was to determine the PPV of the G25.0 code.

**Methods:**

Patients in a tertiary health system with a primary care encounter associated with ICD-10-CM code G25.0 in 2022 underwent medical record review to determine if the consensus criteria from the International Parkinson and Movement Disorder Society for an ET diagnosis were met.

**Results:**

442 patients were included. The PPV of G25.0 in identifying probable ET cases was 74.7% (95% confidence interval (CI) 70.4–78.5%). Among patients prescribed propranolol, the PPV improved to 87.8% (95% CI 78.0–93.6%).

**Discussion:**

Compared to the ICD-9-CM code 333.1, G25.0 is superior for identifying ET cases. A potential limitation of this study is that the consensus criteria applied relies on nonspecific physical exam findings which may lead to an overestimation of the PPV of G25.0.

**Highlights:**

The ICD-10-CM diagnosis code for essential tremor has not been previously validated. The objective of this study was to determine the PPV of the G25.0 code. The PPV in identifying essential tremor cases was 74.7%. The PPV improved among patients prescribed propranolol.

## Introduction

Essential tremor (ET) is the most common movement disorder worldwide with a prevalence as high as 5% in elderly populations [1]. ET can be severely disabling with motor symptoms significantly impeding patients’ activities of daily living and overall quality of life. Studies of nationwide claims databases have additionally shown that patients with ET have higher healthcare utilization and expenditures compared to matched control patients without ET [2,3]. These studies have relied on the *International Classification of Diseases, Tenth Revision – Clinical Modification* (ICD-10-CM) diagnostic code G25.0 “essential tremor” to identify patients with ET, however, the positive predictive value (PPV) of this code has not yet been determined, creating uncertainty surrounding conclusions drawn from such studies.

Louis et al. previously investigated the ability of the *International Classification of Diseases, Ninth Revision—Clinical Modification* (ICD-9-CM) code 333.1 “essential and other specified forms of tremor” to accurately identify patients with ET [4]. The cohort of patients analyzed in this study had a billing record from the Neurological Institute of New York, Columbia University Medical Center associated with the 333.1 code as the primary diagnosis from August 2000 to June 2006. Per the study’s inclusion criteria, medical records of 964 patients were reviewed by a movement disorders neurologist to determine if the clinical criteria for an ET diagnosis were satisfied. Based on this review, the PPV of the ICD-9-CM code 333.1 was 49.0%. When patients with co-existing diagnostic codes for Parkinson’s disease, secondary parkinsonism, multiple system atrophy, or dystonia were excluded, the PPV improved to 57.8%. The generalizability of these findings, however, is limited by patient selection from neurologic specialty clinics rather than primary care or other healthcare settings. Despite this limitation, the study provided important insights into the unsatisfactory performance of the 333.1 code in identifying true ET cases.

The suboptimal accuracy of the ICD-9-CM code 333.1 in identifying ET cases could potentially be explained by the broadness of the coding category. In contrast, the ICD-10-CM G25.0 code is specific to ET. This disease-specific code should ideally improve the ability of researchers to identify ET cases in administrative databases more accurately [5]. However, a study validating the ability of the G25.0 code to identify patients with ET has not yet been conducted. This information is critical for conducting and interpreting the value of claims-based research including ET populations. The present study aimed to determine the PPV of the G25.0 code in identifying true cases of ET.

## Methods

This is a retrospective cohort study of patients who had an outpatient primary care encounter associated with the G25.0 code within the University of Pennsylvania Health System from January 1, 2022, through December 31, 2022.

### Data Source and Population

A query of the electronic medical record (EMR) was performed to identify potential subjects. The included patients were at least 18 years of age and had at least one primary care encounter associated with the ICD-10-CM G25.0 code during the study period.

### Clinical Data Review and Case Ascertainment

After patients were identified via EMR query, two investigators (SDH and SS) independently reviewed each patient’s chart to determine whether clinical criteria for an ET diagnosis were met. Cases were classified as follows: 1) probable ET diagnosis, 2) indeterminate ET diagnosis, and 3) incorrect ET diagnosis. Discrepancies were discussed to consensus. The diagnostic criteria for ET were based on the latest consensus statement on the classification of tremors from the Task Force on Tremor of the International Parkinson and Movement Disorder Society [6]. Classification as a probable ET diagnosis required an isolated action tremor syndrome of the bilateral upper limbs in combination with at least one of the following clinical features:

- At least 3 years duration
- With or without tremor in other locations e.g. head, voice
- Absence of other neurological signs such as dystonia, ataxia, or parkinsonism

Absence of the following exclusion criteria was also required for classification as a probable ET diagnosis:

- Isolated focal tremors (voice, head)
- Task- and position-specific tremors
- Sudden onset and stepwise deterioration

Cases were deemed indeterminate if there was insufficient evidence for an ET diagnosis (e.g. if patients met only one of the four clinical features of ET with the absence of any exclusion criteria or other diagnosis better explaining tremor symptoms). Cases were classified as incorrect ET diagnoses if they met diagnostic exclusion criteria for ET. If a diagnosis other than ET more appropriately explained their tremor symptoms, these cases were also classified as incorrect ET diagnoses. These other diagnoses were identified in free text during chart review. Data was collected and managed using REDCap (Research Electronic Data Capture) tools hosted at the University of Pennsylvania [7,8].

### Statistical Analysis

Baseline and demographic characteristics were summarized using descriptive statistics, including medians and interquartile ranges for continuous variables and percentages and frequencies for categorical variables. The primary endpoint of this study was the PPV of the G25.0 code based on the consensus criteria from the International Parkinson and Movement Disorder Society [6]. In determining the PPV, the G25.0 code was treated as the index test, and a probable ET diagnosis, confirmed through chart review, was considered the reference standard. Indeterminate and incorrect ET diagnoses were considered false positives. Secondary analysis was performed to determine the PPV of the G25.0 code among patients prescribed medications commonly used in the treatment of ET, and separately for patients with a surgical history of deep brain stimulation (DBS) or magnetic resonance-guided focused ultrasound (MRgFUS) thalamotomy. Another secondary analysis was performed to determine the PPV of the G25.0 code among patients who had a neurology encounter during the study period. A tertiary endpoint was the PPV of the G25.0 code after patients with diagnoses of Parkinson’s disease and dystonia were excluded [2,4]. Statistical analysis was performed with Stata version 18 (StataCorp, College Station, TX, USA) by SH.

## Results

**Figure 1:**
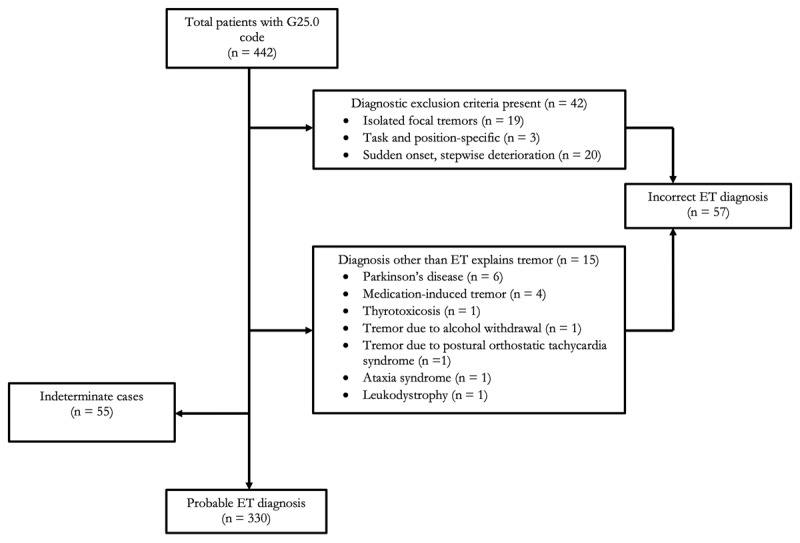
Flow diagram of case classification. Abbreviations: essential tremor (ET).

442 patients met the inclusion criteria for the study. Baseline demographic and clinical information are summarized in Table 1. No patients were excluded from the analysis due to missing data. In the total sample of patients with the G25.0 code, 330 patients (74.7%) were classified as having a probable ET diagnosis, 55 patients (12.4%) had an indeterminate ET diagnosis, and 57 patients (12.9%) had an incorrect ET diagnosis. Figure 1 shows the case classification process.

**Table 1:** **Baseline demographic and clinical characteristics of the sample (total n = 442)**. Abbreviations: body mass index (BMI), deep brain stimulation (DBS), magnetic resonance-guided focused ultrasound (MRgFUS). * Indicates missing data. Six patients lacked BMI data, and one patient lacked race data.


	n (%)

Age, years	*x͂* = 72.5 (63.6–79.4)

Female	251 (56.8)

BMI*	*x͂* = 27.6 (24.5–32.1)

Race*	

White	377 (85.5)

Black	50 (11.3)

Asian	5 (1.1)

Other or Unknown	9 (2.0)

Co-morbidities	

Diabetes	88 (19.9)

Hypertension	240 (54.3)

Hyperlipidemia	266 (60.2)

Depression	79 (17.9)

Anxiety disorder	113 (25.6)

Alcohol use disorder	13 (2.7)

Medications Prescribed	

Propranolol	74 (16.7)

Primidone	26 (5.9)

Gabapentin	32 (7.2)

Benzodiazepine	52 (11.8)

Topiramate	5 (1.1)

Botulinum toxin	4 (0.9)

Surgical History	

DBS	6 (1.4)

MRgFUS thalamotomy	5 (1.1)


Table 2 summarizes the PPV of the G25.0 code in the total patient population as well as in samples selected based on medication prescriptions. Among patients who had a neurology encounter during the study period (n = 85), the PPV was 76.5% (95% CI 66.2–84.4%). Among patients who had a history of either DBS or MRgFUS thalamotomy for ET (n = 11), the PPV was 90.9% (95% CI 49.1–99.0%). After excluding patients with a diagnosis of Parkinson’s disease (n = 17) or dystonia (n = 6), the PPV was 77.4% (95% CI 73.1–81.2%).

**Table 2:** **Positive predictive value (PPV) of the G25.0 code**. Abbreviations: confidence interval (CI).


SAMPLE (n)	PPV % (95% CI)

Total patient population (442)	74.7 (70.4–78.5)

Patients with propranolol prescription (74)	87.8 (78.0–93.6)

Patients with benzodiazepine prescription (52)	75.0 (61.2–85.1)

Patients with gabapentin prescription (32)	81.3 (63.2–91.6)

Patients with primidone prescription (26)	92.3 (72.5–98.2)


Table 3 compares the demographics and comorbid conditions between the incorrect and probable ET diagnosis groups. The only significant difference between the two groups was the frequency of Parkinson’s disease, which was greater in the incorrect diagnosis group (16 (28.1%) vs. 4 (1.2%), p < 0.001).

**Table 3:** **Comparison of demographic characteristics and co-morbidities between incorrect essential tremor diagnosis (n = 57) and the probable essential tremor diagnosis (n = 330) groups**. Continuous variables are reported as medians with interquartile range. Chi-square tests were used to compare categorical variables and the Wilcoxon rank-sum test was used to compare continuous variables. Abbreviations: body mass index (BMI). * Indicates missing data. Two patients in the incorrect diagnosis and three patients in the probable diagnosis group lacked BMI data. One patient in the probable diagnosis group lacked race data.


	INCORRECT DIAGNOSIS n (%)	PROBABLE DIAGNOSIS n (%)	*p*–VALUE

Age, years	*x͂* = 71.4 (65.2–80.5)	*x͂* = 72.9 (65.4–80.0)	0.91

Female	35 (61.4)	181 (54.8)	0.36

BMI*	*x͂* = 26.5 (23.6–31.8)	*x͂* = 27.7 (24.5–31.9)	0.26

Race*			0.82

White	48 (84.2)	279 (84.8)	

Black	8 (14.0)	39 (11.9)	

Asian	0	4 (1.2)	

Other or Unknown	1 (1.8)	7 (2.1)	

Co-morbidities			

Diabetes	9 (15.8)	73 (22.1)	0.28

Hypertension	30 (52.6)	185 (56.1)	0.63

Hyperlipidemia	31 (54.4)	199 (60.3)	0.40

Hyperthyroidism	0	2 (0.6)	0.56

Depression	8 (14.0)	66 (20.0)	0.29

Anxiety disorder	16 (28.1)	81 (24.5)	0.57

Alcohol use disorder	2 (3.5)	7 (2.1)	0.52

Multiple sclerosis	0	2 (0.6)	0.56

Parkinson’s disease	16 (28.1)	4 (1.2)	<0.001

Dystonia	2 (3.5)	4 (1.2)	0.19


## Discussion

Compared to the ICD-9-CM code, the ICD-10-CM code G25.0 for ET demonstrates an improved ability to identify probable cases of ET. Moreover, the PPV of the G25.0 code increases among patients with a prescription for propranolol, the only medication for ET with Food and Drug Administration (FDA) approval [9]. The PPV of the G25.0 code also increases among patients with a primidone prescription, however, there was a smaller number of patients with a primidone prescription compared to propranolol. Understanding the PPV of a diagnostic code is critical to accurately interpreting the results of studies reliant on diagnostic codes for the identification of ET cohorts.

An important consideration in interpreting the results of this study is that the rate of ET diagnosis can be impacted by which set of diagnostic criteria are applied [10]. Differentiating ET from other diagnoses such as Parkinson’s disease or dystonia can be nuanced and challenging, and, historically, ET has been overdiagnosed [11,12,13,14]. Among 104 patients who presented to a movement disorder neurologist with the pre-evaluation diagnosis of ET, only 45.2% had a true diagnosis of ET [12]. The International Parkinson and Movement Disorder Society consensus criteria were used in our study and have notable limitations [6]. The consensus criteria rely on the presence of action tremor in the upper limbs but do not specify the tremor severity required for an ET diagnosis or which tasks must be affected by tremor. The absence of signs of dystonia, ataxia, or parkinsonism is included in the consensus criteria, however, these signs are not listed in detail or specified. The use of diagnostic criteria with more stringent physical examination requirements likely would have resulted in a lower number of patients being classified as probable ET in this study. For example, the Washington Heights-Inwood Genetic Study of ET (WHIGET) criteria incorporate tremor severity and a neurological examination with tasks including pouring water, using a spoon to drink water, finger-to-nose testing, and drawing a spiral [15,16]. When 36 subjects classified as having definite or probable ET per WHIGET criteria were evaluated based on 20 alternative published ET diagnostic criteria, the percentage classified as having ET ranged from 14% to 97% [10]. This wide variability illustrates the importance of evaluating the diagnostic criteria used. The consensus criteria used in our study lacks detailed neurological examination requirements and is likely to have led to a considerable overestimation of the PPV of the G25.0 code.

Validation studies of neurologic conditions have reported variable accuracy of diagnostic code-based case definitions, highlighting the need for continued evaluation of disease-specific diagnostic criteria [17]. In a similar retrospective chart review study, diagnostic codes for drug-resistant epilepsy (ICD-9-CM or ICD-10-CM including both focal and generalized epilepsies) demonstrated a PPV of 0.61 (95% CI 0.53–0.69) [18]. The study’s most sensitive method of case identification, however, added the criterion of at least three antiseizure medications. While the most specific disease-identifying method added the criterion of at least one antiseizure medication other than gabapentin or pregabalin. The authors discuss how the method of case identification should be selected based on the purpose of the study. This concept and use of sensitivity-based analyses when applying case definitions to claims data is critical across neurologic disorders, including ET.

As previously noted, studies of nationwide claims databases have reported that patients with ET have higher healthcare utilization and expenditures compared to matched control patients without ET [2,3]. In an analysis of 2016 Medicare beneficiaries, 27,081 beneficiaries with at least one claim linked to ET had more healthcare visits of all types compared to 27,081 propensity-matched control beneficiaries without ET [2]. Across all healthcare setting types, ET patients had $1068 (95% CI $981– $1154) in additional healthcare payments compared to control patients. This surplus cost per patient may seem modest, but depending on the prevalence estimate of ET this figure translates to an estimated $1.5 billion to $5.4 billion in additional annual Medicare expenditures. In an analysis of Aetna’s administrative claims data from 2017 to 2019 (inpatient and outpatient medical and pharmacy claims), patients with ET had higher total all-cause healthcare costs compared to controls without ET matched based on age, gender, payer type, and ZIP code ($17560 per patient per year (SD $39972) vs. $13237 (SD $27098)) [3]. These results reinforce the findings of the Medicare analysis using a different source of insurance claims data, confirming that ET patients have overall higher medical expenditures. The reason for the higher rate of healthcare use among patients with ET is not fully explained by these studies. Dai et al found that patients with ET had a higher mean number of comorbid conditions compared to patients without ET (5.26 SD (SD 3.21) vs. 4.03 (SD 3.27), *p* < 0.0001) [3]. If the overall burden of illness is higher among ET patients compared to the general population, this could certainly lead to increased healthcare use and costs. Both studies identified patients with ET using the G25.0 code and excluded patients with a co-existing Parkinson’s disease diagnosis. In our comparison of demographic characteristics and co-morbidities between the probable diagnosis and incorrect ET diagnosis groups, the only significant difference identified was a higher rate of Parkinson’s disease within the incorrect diagnosis group. This finding in our study provides support for the methodology of previous claims-based studies that excluded patients with co-existing Parkinson’s disease. Additionally, our results indicate that adding propanol use into the patient selection algorithm could increase case identification accuracy.

In addition to the aforementioned limitations of the consensus criteria, the major limitations of this study are the reliance on EMR and appropriate clinical documentation to categorize cases. ET is a clinical diagnosis that requires a physical examination. It could be the case that patients deemed to have an indeterminate diagnosis simply lacked documentation of their symptoms. The term “probable diagnosis” was used deliberately given that a more definitive determination of diagnosis cannot be made via chart review. This study was also conducted within a single healthcare system; however, a G25.0 code within a primary care setting was used as inclusion criteria to increase the generalizability of the findings. If patients were selected based on a G25.0 code associated with a neurology encounter, the PPV likely would have been higher. The sensitivity analysis among the patients who did have a neurology encounter during the study period did show the PPV of the G25.0 code was slightly higher.

In summary, this study provides a valuable estimate of the ability of the G25.0 code to identify probable ET cases. While acknowledging that the non-specific nature and lack of detail in the consensus criteria may have overestimated the PPV of the G25.0 code, the results of this study can be used to increase the quality of future research on ET populations. This information can aid in optimizing inclusion and exclusion criteria for ET cohorts and improve the validity of results based on the analysis of claims data.

## Financial Disclosures

In the past three years, Dr. Farrar has received compensation for serving on two NIH data safety monitoring boards and advisory boards or consulting on clinical trial methods from Vertex Pharma and EicOsis Pharma. The other authors have no financial disclosures.
